# Developing Children’s Creativity and Social-Emotional Competencies through Play: Summary of Twenty Years of Findings of the Evidence-Based Interventions “Game Program”

**DOI:** 10.3390/jintelligence10040077

**Published:** 2022-10-02

**Authors:** Maite Garaigordobil, Laura Berrueco, Macarena-Paz Celume

**Affiliations:** 1 Faculty of Psychology, University of the Basque Country, 48940 Leioa, Spain; 2 Laboratoire de Psychologie et d’Ergonomie Appliquées, University Paris-Cité, 92100 Boulogne-Billancourt, France

**Keywords:** childhood, social development, emotional development, intelligence, creativity, cooperative games, intervention programs

## Abstract

This work presents the results of four cooperative-creative game programs (Game Programs). In all four studies, experimental designs with repeated pretest-posttest measures and control groups were used. Validation samples ranged from 86 to 178 participants, randomly assigning participants to the experimental and control conditions. Before and after each program, a battery of assessment instruments was applied to measure the variables under study. The intervention consists of conducting a weekly game session during the school year. The results of the posttest covariance analyses confirmed a significant impact: (1) in social development, by increasing various positive social behaviors and decreasing many negative social behaviors; by increasing assertive cognitive strategies and prosocial resolution of interpersonal problems; and by enhancing relationships and positive communication among group members; (2) in emotional development, by improving self-concept, peer image, and emotional stability; and (3) in cognitive development, by increasing verbal intelligence, verbal and graphic-figurative creativity, as well as creative personality behaviors and traits. This work provides empirical evidence of the relevance of cooperative-creative play in child development.

## 1. Introduction

### 1.1. Theoretical Foundation of the Game Programs: Contributions of Play to Child Development

Play is inherent to a child’s very nature, and psychologists, philosophers, and pedagogues have referred to its importance for the development of personality. From the conclusions of the studies, it is clear that “play, that activity par excellence of childhood, is a vital and indispensable activity for human development, as it contributes relevantly to psychomotor, intellectual, social, and affective-emotional development” (Garaigordobil 2003a, p. 97). From different epistemological models, pioneering theorists and researchers in play research have emphasized the relevant role of play in psychomotor development (Piaget [1945] 1979; Wallon [1941] 1980), in the development of intelligence and creativity (Piaget [1945] 1979; Vygotski [1932] 1979, 1982; Wallon [1941] 1980; Winnicott [1971] 1982), in the social development (Elkonin [1978] 1980; Vygotski 1982), and in affective-emotional development (Freud [1920] 1981; Winnicott [1971] 1982). Although authors such as Piaget, Wallon, Vygotsky, Elkonin, and Winnicott were pioneers in the formulation of the relevance of play in human development, among others, recent experimental, correlational, and observational studies have ratified the effectiveness of play in the development of motor skills (Carpio et al. 2020), in language development (Ramos and Allcca 2021), in moral development (Davis and Bergen 2014), in social competencies (Villavicencio 2019), and in emotional competencies (Zych et al. 2016).

Various observational, experimental, and correlational studies (Lavega et al. 2014; Navarro 2002) have confirmed the relevance of motor play for physical and sensory development. The variety of games that children play from an early age are a fundamental stimulus for their psychomotor development, i.e., motor coordination (the global coordination of all parts of the body, balance, eye-hand and eye-foot coordination, precision of movements, muscle strength, etc.) and perceptual structuring (visual, auditory, rhythmic, temporal, and tactile perception, spatial orientation, etc.). In summary, psychomotor play permits the development of the body and senses (Garaigordobil 1999a, 2003a, 2009; Shingjergji 2020; Votadoro 2001).

Concerning the development of intelligence and creativity, scholars of the subject have underlined the relevant role of children’s play in the evolution of thinking that leads to abstraction, as well as in the development of creativity (e.g., Berretta and Privette 1990; Chen Tsai 2012; Karwowski and Soszynski 2008; Russ and Schafer 2006). Regarding social development, much research has confirmed the relevance of play for socialization, especially for the acquisition of social knowledge and social communication skills (e.g., Barnes 2014; Lillard 2017; Mellou 1994). A large part of children’s social interactions throughout childhood takes place in the context of play, where they establish contact and relationships with other children. All the ludic group activities that children carry out throughout childhood stimulate the progressive development of what we call the “Social Self”. Studies carried out with collective symbolic games of representation (e.g., Celume et al. 2020), also called dramatic or sociodramatic play or role-playing, conclude that these games have intrinsic qualities that make them very relevant in the process of child socialization, which are activities of inestimable value for children’s development and social adaptation.

Other observations relate to affective-emotional development, where play is seen as an activity that allows achieving psychological balance in the first years of life. Play provides the opportunity to express negative feelings, experiment with alternative solutions, process new information without the consequences it would have in real life, resolve affective crises, and express/control emotions (e.g., García-Huidobro 2004). Difficulties are part of the process of growth in every individual. If children are to overcome the successive phases, they must be allowed to express their fantasies, desires, fears, and interpretations, and this opportunity is presented in play. “Play promotes affective-emotional development, psychic balance, and mental health” (Garaigordobil 2003a, p. 93).

Play is an instrument of expression and emotional control, essential for growth. This activity contributes to the integration of the personality because children play for pleasure, to express aggressiveness, to master their anxiety, to increase their experiences, to establish social contacts, etc. Play has a vital role in children’s affective-emotional balance, as it enables the expression and release of childhood tensions. Various cognitive and emotional processes are deployed in the symbolic game of representation, developing children’s creative thinking and key emotional competencies (Celume et al. 2019a). The dialogical and protected space that promotes play allows children to try different ways of being and acting and thus to develop cognitive processes that influence their creativity and their affective-emotional well-being (Celume et al. 2019b).

Recapitulating the contributions of play to children’s integral development, we can confirm that play is a vital activity for human development because the ludic activities that children perform throughout childhood allow them to develop their thinking, meet needs, elaborate traumatic experiences, discharge tensions, explore and discover the joy of creating, fulfill their fantasies, reproduce their acquisitions by assimilating them, relate to others, broaden their horizons, etc. Therefore, it can be said that stimulating play is synonymous with promoting child development, and this activity also has a significant preventive and therapeutic function. A recent review (Garaigordobil 2022) of the effects of play in child development showed that the results of research on the relevance of play have currently driven many professionals of psychology and education to emphasize the inclusion of play in educational and therapeutic contexts. The main findings of this review state that play impacts several levels of child development such as (I) physical and sensory development, (II) intelligence and creativity, (III) social development, and (IV) affective-emotional development. We describe these main findings below.

(I) Physical and sensory development: Thanks to the movement games that children play from early ages (games with the body, with objects, and with others; for example, playing with a ball, hoops, stilts, and roller skates, building with blocks, modeling with mud or plasticine, jumping rope, jumping with an elastic rope, swinging, riding tricycles and bikes, etc.), children can discover new sensations; progressively coordinate the movements of their body, which become more refined, more precise, and more effective; structure the mental representation of the bodily schema, the “I-body”, which is the first “brick” in the construction of personality; discover themselves at the origin of the material modifications they produce when they build or model; explore and expand their sensory and motor skills; conquer their own body and the outside world; have mastery experiences that foster self-confidence; and more importantly, they obtain intense pleasure.

(II) Intelligence and creativity: Motor and sensory manipulative games are an instrument for the development of motor thinking. Play, in addition to being a source of learning, opens up areas of potential development. In play, attention and memory are doubled. Representative play fosters cognitive decentralization, which drives the development of symbolic thinking, or representative intelligence. Symbolic play encourages fantasy–reality discrimination. Social play facilitates language development. Play promotes the development of higher psychological functions because fiction is a necessary avenue for the development of abstract thinking. Play originates and develops imagination and creativity.

(III) Social development: Games of representation stimulate the development of positive social behaviors (helping, cooperating, sharing, negotiating, etc.) and decrease the negative ones (aggressiveness). In games of representation, children discover the social world of adults, the rules that govern these relationships, and how to prepare for work. Games of representation promote moral development because they are a school of self-control and will and an assimilation of norms of conduct. Games of representation foster various social competences (social skills, group cohesion, problem-solving, etc.). In addition, studies that have analyzed the effects of rule games (structured around objective rules whose transgression constitutes a fault), i.e., games of sensorimotor combinations (races, throwing regulated marbles) or intellectual games (board games, cards, chess, checkers, Parcheesi, dominoes, tic-tac-toe), conclude that, in these games, children develop strategies of social interaction and learn to control aggressiveness because the rules regulate the control of behavior, and they are a school of responsibility and democracy.

(IV) Affective-emotional development: Play is a pleasurable activity that generates emotional satisfaction and feelings of happiness. Symbolic play, games of representation, is an activity that allows the control of the anxiety generated by difficult and traumatic experiences. Play is an activity that allows the control of the anxiety generated by internal sources, aggressiveness, and child sexuality. Symbolic play, games of representation, is the performance of forbidden and/or unattainable desires. Play encourages the expression of emotions and facilitates emotion regulation. The game of representation facilitates the progressive process of psychosexual identification. Play is a means of learning conflict-resolution techniques. Play in general improves self-esteem, is a means of self-affirmation, is proof of children’s personalities, and is an early source of a sense of identity. Symbolic play, games of representation, enhances the capacity for empathy. Play has a significant therapeutic value.

### 1.2. Cooperative Games: Benefits for Child Development

Cooperative games are defined as games in which the players give and receive help to contribute to a common end. These games stimulate communication, cohesion, and trust, and they are based on the idea of accepting, cooperating and sharing, and playing and inventing together (Orlick 1990). Within this category of games, games of representation are included (e.g., representing scenes in which each player contributes with their role), but they also include motor games (e.g., using the bodies of several players to construct a snake that crawls on the ground, or a train that goes on a journey) and rule games (e.g., burnt field cooperative, in which one team’s burnt members go over to the other team and keep on playing when they are “touched (burnt) by the ball”).

According to Garaigordobil (2003a), cooperative games have five features. (1) Participation: as everyone participates in these games, there are no eliminated members, nor does anyone lose; the goal is to participate to achieve group goals. (2) Communication and friendly interaction: because these games structure group communication processes that involve listening, dialoguing, making decisions, negotiating, etc., these games stimulate a friendly multidirectional interaction between players, the expression of positive feelings toward others, etc. (3) Cooperation: as they enhance relational dynamics that necessarily lead the players to help each other to contribute to a common goal, each player contributes with their role, full of meaning and necessary for the achievement of the game, a situation that generates a feeling of acceptance in each person, which positively influences their self-concept and others’ image. In these games, the players’ objectives are closely linked in such a way that each one can only achieve their goals if the others reach their own goals. The results that each player pursues are therefore beneficial for the rest of the teammates with whom they are interacting. This is the opposite of competitive games, in which the objectives of the players are related, but exclusively, and a participant only reaches their goal if the others do not achieve it. (4) Fiction and creation: because, in many cooperative games, children act “as if”: as if they were snakes, turtles, bells, blind, trains, machines, etc., and represent real or fantasy scenes, etc., as well as combine stimuli to create something new (e.g., joining different objects to create musical instruments, etc.). (5) Fun: because when playing cooperatively, the players have fun interacting with their peers in a positive, friendly, and constructive way.

Since the 1970s, observational and experimental studies have proven the benefits of cooperative play in child development. The results of the review of these studies can be seen in Table 1.

In addition, research comparing the differential impact of cooperative and competitive games confirms the advantages of cooperation. Some studies (Bay-Hinitz et al. 1994; Bay-Hinitz and Wilson 2005) confirmed that, during cooperative games, cooperative behaviors increase and aggressive behaviors decrease, whereas after implementing a competitive game program, aggressive behaviors increase and cooperative behaviors decrease. Finlinson et al. (2000) found that positive social behaviors were higher during cooperative games, whereas more negative behaviors were observed in competitive games. A higher level of negotiation strategies and sharing behaviors among group members has also been found in cooperative versus competitive games (Zan and Hildebrandt 2003). Recently, Eriksson et al. (2021) highlighted that cooperative and competitive board games led to the same amount of cooperative and prosocial behaviors. Participants competed more after playing competitive games, but they enjoyed themselves more in cooperative games and preferred them.

In summary, previous studies have shown the effects of cooperative games on variables of social, emotional, and psychomotor development, although few have shown the effects on variables related to intelligence and creativity. Therefore, this work aimed to show the positive effects of “Game Programs”, cooperative play programs, in all the dimensions of child development. The empirical studies carried out with the four intervention programs had three main objectives: (1) to design the intervention programs configured with cooperative-creative games targeting different age groups; (2) to carry out an experimental implementation of the programs that consists of applying a weekly intervention session during an entire school year; and (3) to evaluate the effects of these cooperative experiences on factors of social, affective-emotional, and psychomotor development, as well as on intelligence and creativity.

The current work summarizes the effects of four psychoeducational intervention programs based on the research question about how cooperative-creative play may have significant benefits for child development. Throughout the last two decades, different studies were carried out to test the significance of results regarding competency development, and in this current work, we proposed to review these findings, offering a global perspective of the outcomes and postulating that cooperative-creative games have relevant benefits for several variables of socio-emotional development, as well as for the development of intelligence and creativity. The programs designed and evaluated in the previous work and summarized in the current manuscript contain games with two structural components: cooperation and creativity. On the one hand, they are configured with cooperative games, i.e., games that consist of giving and receiving help to contribute to a common goal and to achieve team goals with the necessary contribution of all players, and, on the other hand, they are configured with creative games that structurally contain dynamics that activate verbal, graphic-figurative, plastic-constructive, and/or dramatic creativity.

## 2. Materials and Methods

### 2.1. Participants

Validation samples in each of the four programs were chosen randomly, and participants were randomly assigned to the experimental and control conditions. In the Game Program 4–6 years (Garaigordobil 2007), there were 53 experimental (3 groups) and 33 control participants (2 groups) (47 boys; 39 girls). In the Game Program 6–8 years (Garaigordobil 2005a), there were 125 experimental (6 groups) and 53 control participants (2 groups) (93 boys; 85 girls). In the Game Program 8–10 years (Garaigordobil 2003b), there were 126 experimental (4 groups) and 28 control participants (2 groups) (86 boys; 68 girls). In the Game Program 10–12 years (Garaigordobil 2004a), there were 54 experimental (2 groups) and 32 control participants (2 groups) (34 boys; 52 girls).

### 2.2. Procedure

The four Game Programs were evaluated experimentally and are evidence based. In order to measure the effects of each program on child development, successive investigations were proposed using the experimental designs of repeated pretest-posttest measures with control groups.

After selecting the educational centers, in the four studies carried out to evaluate the effects of each program, a set of evaluation instruments was administered before and after applying each program (September and June) to measure different development variables that the program was hypothesized to affect. The groups were randomly assigned to the experimental or control conditions. The experimental participants performed an experimental implementation of the programs, consisting of receiving a weekly intervention session during an entire school year. The duration of the sessions was variable: 75 min in preschool (4–6 years), 90 min in the first and second cycle of Primary Education (6–10 years), and 120 min in the program of the last cycle of Primary Education (10–12 years).

To avoid the Hawthorne effect, the control participants carried out the curricular activities of their school program, thereby receiving a different type of instruction and the same level of attention. They carried out the activities of the tutorials that the educational centers had detailed in their educational projects. In these tutorials, for example, they talk about the problems that may exist in the group, about issues related to social interactions or emotions, etc. Therefore, it is unlikely that the Hawthorne effect was produced in the control participants, since during the time that the experimental ones were carrying out the cooperative-creative game program, the control ones were carrying out tutoring activities, which offered them the same level of attention.

The study met the ethical values required in research with human beings, following the fundamental principles included in the Declaration of Helsinki, its updates, and current regulations (informed consent and right to information, protection of personal data, and guarantees of confidentiality, nondiscrimination, gratuity, and freedom to leave the study at any stage). The participants and their parents were informed of the study. Consent was obtained from all parents of the participants involved in the research. Parents had to inform if they did not want to participate in the study (passive consent). All parents accepted the participation of their children, largely due to the expectation of the benefits that the programs could have on their children’s development. The children were informed and also had the opportunity not to participate; however, all the children accepted to participate in the cooperative play sessions.

### 2.3. Instruments

To evaluate the effects of the four programs before and after each intervention, several evaluation instruments, with psychometric guarantees of reliability and validity, were administered to measure the dependent variables that the program was hypothesized to affect. The variables evaluated and the instruments used are presented in Table 2 for preschool children (4–6 years), Table 3 for children 6–8 years old, Table 4 concerning children 8–10 years old, and finally Table 5 gathers the quantitative instruments used for measuring 10–12-year-old children’s competencies. As can be seen in these tables, many of the variables evaluated are related to emotional intelligence, although intelligence and creativity variables are also included. The tables displayed below present the quantitative instruments used for the evaluation of the programs and are presented here as a means to describe the instruments used. Below the tables, we present a qualitative review of the main instrument used as the independent variable, the game program, presenting the similarities of all four programs.

### 2.4. The Intervention: Game Programs, a Cooperative-Creative Game Proposal to Promote Child Development

Taking previous works as a reference, a line of psychoeducational intervention configured with four cooperative and creative game programs, the Game Programs were systematized. These programs, which targeted children in early childhood (pre-school) and Primary Education (the 4–6 years program (Garaigordobil 2007), the 6–8 years program (Garaigordobil 2005a), the 8–10 years program (Garaigordobil 2003b), and the 10–12 years program (Garaigordobil 2004a) aim to promote child development. The practical proposals for each age group have a theoretical foundation manual that reflects on the contributions of play, prosocial behavior, and creativity in human development (Garaigordobil 2003a).

These programs have three main general objectives: (1) prevention, as this experience attempts to prevent developmental problems; (2) development, as these experiences are intended to enhance the integral development of children who do not present difficulties in their growth, focusing primarily on various socio-emotional aspects and creativity; and (3) therapy because, through these games, we try to socially integrate children who have difficulties in their social, emotional, or intellectual development. On a more specific level, the Game Programs propose the following concrete objectives.

They promote social development by stimulating children’s knowledge of each other, increased multidirectional, friendly, positive, and constructive interactions with their group peers, and group participation; friendly intra-group relationships; verbal and nonverbal communication skills, such as presenting, actively listening, dialoguing, negotiating, and making decisions by consensus; an increase in social behaviors that facilitate socialization (leadership behaviors, joviality, social sensitivity, respect-self-control), as well as a decrease in disruptive socialization behaviors (aggressive-stubborn behaviors, apathy-withdrawal, anxiety-shyness, and antisocial behaviors); prosocial behavior (behaviors such as giving, helping, cooperating, sharing, comforting); and moral development, such as accepting social norms implicit in the instructions of the games (taking turns, cooperation, roles, values, dialogue, tolerance, equality, solidarity, etc.).

The Game Programs promote affective-emotional development by fomenting the identification of various emotions; the understanding of various causes of positive and negative emotions, as well as the consequences thereof; the expression of emotions through dramatization, activities with music-movement, and drawing and painting; coping with or the resolve of negative emotions; empathy for the emotional states of other human beings; emotional stability; self-concept-self-esteem; and feelings of pleasure and subjective psychological well-being.

They stimulate the development of factors of intelligence and creativity, such as attention; memory; the capacity for symbolization; verbal logical reasoning; and verbal creativity (language), graphic-figurative creativity (drawing/painting), plastic-constructive creativity (construction of objects), and dramatic creativity (representation of roles).

As previously explained in the introduction, games within the program stimulate communication, cohesion, trust, and the development of creativity underpinned by the idea of acceptance, cooperation and sharing, playing, and inventing together. This is conducted through the following 5 essential points: (1) everybody’s participation; (2) dialogue and active communication; (3) mutual cooperation; (4) fiction and creation; and (5) fun. Programs contain various types of games and use various group dynamic techniques for the development of action and other techniques to stimulate and regulate discussion.

The Game Programs contain a wide range of cooperative games that stimulate prosocial behavior (helping behaviors, trust, cooperation) and creativity (verbal, graphic-figurative, plastic-constructive, and dramatic). Many programs and game books were reviewed to design these game proposals. Some of the activities included were adapted from other programs that had similar objectives. For example, some are games of Orlick ([1978] 1986, 1990) pioneering experiences with cooperative games, others are traditional games that met the requirements of cooperative play, and still others were originally competitive games that were transformed into cooperative games by changing their rules (e.g., the game of eliminatory chairs eliminates children; in its cooperative version, only chairs are removed, and all the players sit on the remaining chairs), and other games were invented and created specifically for these programs.

In the implementation of the program, a weekly game session is carried out throughout the school year of variable duration, 75 min in preschool, 90 min in the first and second cycle of Primary Education (6–10 years), and 120 min in the last cycle of Primary Education (10–12 years). The experience takes place on the same day, on the same weekly schedule, and in the same physical space (a large classroom free of obstacles). The same adults always direct the sessions, including the teacher of the group, who directs the dynamics of the game, and an adult with psycho-pedagogical training (psychologist), who carries out the assessment before and after implementing the program and the systematic observation of the game sessions.

The organization of a game session was generally structured with a sequence of 2–4 cooperative-creative recreational activities and their subsequent debates. The session was organized around three phases, led by the group’s regular teacher.

Opening phase: The sessions begin with the group members sitting in a circle on the floor. In this spatial position, the objectives of cooperative and creative games are briefly discussed: having fun, making friends, learning to help each other, learning to collaborate with classmates to perform things as a team, learning to listen to each other, be respectful of others’ ideas, and be original, creative, imaginative, etc. Usually, the adult communicates the opening of the cooperative play session to the group, and, in the first sessions, he/she informs them about the general objectives of the experience, but after 2 or 3 sessions, he/she asks the group about its objectives to encourage two-way communication (feedback) about the objectives of the game program.

Development phase of the game sequence: In this phase, the 2, 3, or 4 games that make up the session are carried out successively, with the following procedure for each recreational activity. (1) The adult presents the instructions of the game to be played, and the members of the group play the game following the instructions. (2) After each game is played, a discussion takes place related to the actions and interactions that occurred in the game. For this purpose, the players sit on the floor in a circle, and the adult asks questions related to the objectives of the program, the specific objectives of the game performed, or what he/she observed during the game (for example, communication, participation, cooperation, originality of the ludic products, conflicts, resolutions provided, etc.).

Closing phase: After performing the game sequence, the closing phase begins, in which reflection and dialogue about what happened during the game session (feelings experienced, participation, rejections, respect for the rules, cooperation, etc.) take place. The adult leading the intervention asks the group members how they felt during the game session and whether they want to comment on any aspect of the session that was not addressed in the dialogue or debate phases after each game. Closure is an exercise of reflection during which the players verbalize the positive aspects of the experience, as well as the problems that arose and the solutions to them. Thus, its role in the children’s cognitive-moral development is important. In this phase, the adult, besides promoting communication within the group about the experience, provides social reinforcement, the verbal assessment of the helping behaviors, dialogue, or cooperation observed, emphasizing the value and creativity of the products elaborated in the activities involved in this process.

To clarify the type of activities contained in the Game Programs, three games are described below, informing about the specific developmental functions they stimulate.

Cooperative drawings: Teams of 4 players are formed, and each player receives one-half of a sheet of white cardboard and a pencil. First, each team member makes a drawing for 10 min, for example, a house. Then, the adult says, “pass the drawing to the right”, and each player gives the drawing they have made to their classmate on the right. Now, they have 5 min to add elements to their classmate’s drawing; for example, to the drawing of the house, the player adds a tree. The operation of passing the drawing is conducted 3 times, so that all the members of the team add elements to the drawing of their three classmates, resulting in 4 drawings made with everyone’s cooperation. In the last pass, each player receives the drawing that they began and colors it to their liking with the paint box they share. At the end, there is an exhibition with all the drawings, and the participants comment on them. This game encourages non-verbal communication (active listening), group cohesion (feelings of belonging), cooperation (drawing among several people), the pleasure of creating (feelings of achievement and mastery that improve self-concept), feelings of acceptance (each player has a necessary role to perform the game), as well as attention, the ability to symbolize, graphic-figurative creativity, visual-spatial perception, and eye-hand coordination.

Discover the excitement: The group is divided into teams of 6–7 players, to each of whom an emotion is assigned (sadness, love, hate, joy, envy, fear, etc.). The players of each team have 15–20 min to agree on how to represent the situation so that the rest of the players can guess the emotion from the scene. For example, they may express sadness by dramatizing a funeral or joy by representing a birthday. Team members clarify how to represent the emotion, they disguise themselves, and they create some supporting material for the performance. Subsequently, each team performs the representation in turns, and the rest must guess the emotion expressed. This game stimulates verbal and non-verbal communication (active listening and decision-making), cooperation (role representation and coordination with other roles to represent the scene expressing the emotion), group cohesion (feelings of belonging), the pleasure of representing and guessing the emotion expressed, the ability to identify and express emotions through dramatization, feelings of acceptance (each player has a necessary role to perform the game), attention, symbolization, and dramatic creativity.

The new evolution of species: The group is divided into teams, and each team receives a sheet of white cardboard, several photos of animals, scissors, and various glues. The game consists of cooperatively inventing a new animal species. To do this, team members must select photos of animals, and then cut out different anatomical parts (ears, eyes, legs) that they will use to form the body of the new animal. When they have enough cutouts of different body parts of different animals, they begin to paste them onto the cardboard, configuring a new invented animal made up of parts of other existing animals. Then, they invent a name for their animal and make a brief description of it (type of animal: land, sea, air…, what it eats, where it lives…). Finally, an exhibition is held with the animals created and each team presents its animal. This game promotes verbal communication (active listening and decision-making), cooperation (inventing a new animal with everyone’s contribution), group cohesion (feelings of belonging), the pleasure of creating (feelings of achievement and mastery that improve self-concept), feelings of acceptance (each player has a necessary role to perform the game), attention, the capacity for synthesis (configuration of the whole through the integration of parts), plastic-constructive creativity, visual perception, and eye-hand coordination.

## 3. Results

To analyze the effects of the program with the data obtained in the assessment instruments applied in the phases pretest-posttest, we calculated the means and standard deviations of each target variable for the experimental and control participants in the pretest phase, in the posttest phase, and in the differences pretest-posttest. In addition, we conducted univariate analyses of variance (ANOVAs) with the pretest and the posttest scores, comparing the experimental and control conditions. To further probe these effects, taking into account the differences between the two conditions in the pretest phase, analyses of covariance (ANCOVAs) were then performed with the posttest scores (covarying out the pretest scores) on each of the dependent variables, and effect sizes were calculated (Cohen’s *d*: <0.2, small, 0.5 = medium and >0.8 large). To calculate the ANCOVAS, the usual procedure was followed. In each variable, the posttest was introduced as the dependent variable, the pretest score as the covariate, and the condition (experimental and control) as a fixed factor.

The statistical analyses carried out with the pretest-posttest data obtained (descriptive and inferential analyses) in each of the four investigations validated these experiences. The evaluation of the programs for the four age levels ratified numerous positive effects when comparing the change in various development factors in the children who participated in these cooperative-creative game experiences during the school year (experimental group) compared to those who did not have the opportunity to play the games that academic year (control group). The descriptive (means and standard deviations) and inferential (analysis of variance and covariance) results in the pretest and posttest phases and the pretest-posttest difference are presented in Table 6, Table 7, Table 8 and Table 9.

In the evaluation of the program targeting preschool children (Garaigordobil 2007), the results of the posttest covariance analyses (see Table 6) confirmed that this game experience promoted a significant improvement in many variables. From the point of view of social and affective-emotional development, the program stimulated an increase in: (1) altruistic behavior with peers, (2) prosocial strategies for solving interpersonal problems, (3) normativity or knowledge and compliance with social norms indicated by adults, and (4) affective maturity or the ability to give affective responses according to the evolutionary level (tendency). From the point of view of cognitive development, the program promoted an increase in: (1) verbal intelligence (Verbal IQ) and global intelligence (global IQ) (tendency); (2) neuropsychological maturity factors such as verbal fluency; (3) verbal creativity (fluency, flexibility, and originality); (4) graphic-figurative creativity (abreaction, elaboration, fluency, and originality); (5) behaviors and traits of creative personalities evaluated by teachers; and (6) creative thinking associated with the analysis of an image (ability to perceive unusual or strange details, fluency, and originality to identify problems and seek solutions to these problems) (Garaigordobil and Berrueco 2007a, 2011). The effect size was large in the following variables: verbal creativity (flexibility, fluency, originality), graphic creativity (elaboration, fluency, originality), and creative thinking (attention to detail, fluency, originality). A medium effect size was found in the verbal fluency.

In the research on the program for children aged 6 to 8 years (Garaigordobil 2005a), the results of the posttest covariance analyses (see Table 7) revealed significant changes in several variables evaluated. From the point of view of social development, the program promoted (1) an increase in positive social behaviors (prosocial leadership, joviality or joy in social interaction, social sensitivity or helping behaviors, respect for the rules of sociability-self-control of impulses) and a decrease in negative social behaviors (aggressiveness-stubbornness, apathy-withdrawal, anxiety-shyness) in social interactions with peers, improving the general social adaptation of the children in the school context; (2) an increase in the capacity for group cooperation (decrease in the time they need to perform a cooperative task and an increase in cooperative facilitating behaviors such as giving-receiving and helping behaviors); and (3) communication and relationships of acceptance within the group. From the point of view of affective-emotional development, the program stimulated an improvement in: (1) self-concept and (2) emotional stability. In terms of psychomotor and cognitive development, the program facilitated: (1) a greater recognition of the body schema and (2) an increase in various maturity skills for school learning (verbal comprehension, numerical aptitude, perceptual aptitude) and the overall maturity index for school learning (Garaigordobil and Echebarria 1995; Garaigordobil et al. 1996). The effect size was large in the following variables: social behavior (social sensitivity, aggressiveness, apathy withdrawal, anxiety shyness, social adjustment) and group cooperation (task performance time, giving-receiving behaviors, asking-receiving behaviors). A medium effect size was found in many variables, such as social behavior (leadership, joviality, respect for self-control), group cooperation (helping behaviors), body schema, and school learning (verbal comprehension, numerical aptitude, maturity index). The research of the program was presented as a Doctoral Thesis at the University of the Basque Country (Garaigordobil 1994) and was nominated for the “Extraordinary Doctorate Award” granted by the University of the Basque Country.

In the research of the program for children aged 8 to 10 years (Garaigordobil 2003b), the results of the posttest covariance analyses (see Table 8) highlighted a significantly positive impact of the program. In social development, it promoted (1) an increase in altruistic prosocial behavior as the scores in competitive and betrayal behaviors, whose purpose is to achieve greater individual benefits at the expense of harming the other, decreased; (2) a decrease in non-assertive social behaviors (passive and aggressive) in interactions with peers; and (3) an increase in positive messages towards group members (especially socially valued physical characteristics, cognitive skills, prosocial behaviors, skills for various activities, etc.) and a decrease in negative messages (referring to intellectual difficulties, socially depreciated physical characteristics, lack of skills for activities, negative personality characteristics, insults, etc.). With regard to affective-emotional development, the program promoted an improvement (1) of the global self-concept, especially in relation to the social and affective self-concept. In terms of cognitive development, there was also a significant increase in (1) verbal creativity (fluency, flexibility, originality) and (2) graphic-figurative creativity (fluency, flexibility, originality, connectivity, abreaction, fantasy). The effect size was large in the following variables: altruistic behavior, assertive behavior, communication (positive and negative messages), verbal creativity (fluency, flexibility, originality), and graphic creativity (fantasy). A medium effect size was found in many variables such as: social behavior (aggressive, passive, helping), self-concept (social and global), and graphic creativity (fluency, originality, connectivity, abreaction). These results validated the program (Garaigordobil 1995, 1996, 1999b, 2014), and the design and evaluation of the program received the “First National Prize for Educational Research 1994” awarded by the Ministry of Education and Science (Garaigordobil 1996).

The results of the posttest covariance analyses (see Table 9) in the experimental validation of the program for children aged 10 to 12 years (Garaigordobil 2004a) showed a significantly positive effect of the program on a variety of variables. From the point of view of social development, it stimulated (1) an increase in the social behaviors of respect for the rules of sociability and self-control of impulses, social leadership behaviors associated with the spirit of service and popularity; (2) assertive behaviors in interactions with peers; (3) prosocial behaviors (evaluated by parents); (4) the image of the group members perceived as more prosocial and creative; (5) assertive cognitive social interaction strategies; and (6) a decrease in aggressive behaviors in peer interactions and (7) in antisocial and delinquent behaviors. From the point of view of affective-emotional development, the program promoted (1) an increase in emotional stability and (2) an improvement in total and creative self-concept. In cognitive development, a positive impact of the program was confirmed in (1) verbal intelligence (verbal IQ); (2) verbal creativity (originality); (3) graphic-figurative creativity (abreaction, originality, and elaboration); as well as (4) an increase in creative personality behaviors and traits (self-evaluation and evaluation of teachers) (Garaigordobil 2005b, 2005c, 2005d, 2006, 2008). The effect size was large in the following variables: aggressive behavior, assertive social interaction strategies, creative personality (teacher evaluation), verbal creativity (originality), and graphic creativity (abreaction, elaboration, time needed to paint a creative picture). A medium effect size was also found in many variables, such as social behavior, antisocial behavior, delinquent behavior, self-control behavior, prosocial behavior evaluated by parents and peers, self-concept, emotional instability, creative personality (self-assessment), creative behavior (peer evaluation), and the graphic creativity of a picture (originality). The work received the “First National Prize for Educational Research 2003”, awarded by the Ministry of Education and Science (Garaigordobil 2005b).

The results obtained in the successive studies confirmed the positive effects of the Game Programs on various social, emotional, intellectual, and psychomotor factors of child development, validating this line of psychological intervention based on cooperative and creative play for the development of children’s personalities from ages 4 to 12 years. These results provide empirical evidence of the relevance of cooperative-creative play in child development and validate game programs to promote child development in educational, family, and therapeutic contexts.

## 4. Conclusions and Discussion

These results point in the same direction as other studies that confirmed the positive effects of play on various factors of social, emotional, and psychomotor development (Andueza and Lavega 2017; Blazic 1986; Brownell et al. 2002; Carlson 1999; Cuesta Cañadas et al. 2016; Garcia 2021; Guerreros 2021; Guevara and Ubillus 2019; Grineski 1989; Beltrán 2007; Lavega et al. 2014; Navarro-Patón et al. 2019; Mender et al. 1982; Mikami et al. 2005; Miralles et al. 2017; Ochoa 2019; Orlick 1981; Orlick et al. 1978; Orlick and Foley 1979; Valencia 2010; van der Aalsvoort and van der Leeden 2009; Vega 2018; Zanandrea 1998) and also ratify the results of the recent review (Garaigordobil 2022) that shows the positive effects of play on the development of intelligence and creativity.

The work of the design, implementation, and evaluation of the four Game Programs, which have been developed for more than two decades, provides empirical evidence of the contributions of play to integral child development. This line of research allows us to conclude that play, cooperation, and creativity are relevant activities for child development, providing empirical evidence of the important contributions of cooperative-creative play to psychomotor, intellectual, social, and affective-emotional development. In addition, the results ratify the perspective of many psychology and education professionals who emphasize the inclusion of ludic group activities as a preventive and developmental instrument in clinical and educational contexts.

The effects of the programs on the different variables of social, emotional, psychomotor, and intellectual development are derived, on the one hand, from the characteristics of the games themselves and, on the other hand, from the emphasis that is made during the debates of the game sessions. In these phases, we reflect on the satisfaction generated by receiving positive messages about oneself, as well as on the moral damage and its impact on one’s behavior of perceiving negative messages about oneself. It also reflects on the benefits of cooperating versus competing, the difficulties of working in a group, or the pleasure of creating, etc., thereby stimulating cognitive and moral development with their consequent implications on behavior and development. In addition, in these debates, the adult who directs the intervention values and reinforces the prosocial behaviors observed in the course of the session, the expression of feelings, the creativity of the ludic products elaborated cooperatively, etc., and all this promotes positive behavioral, cognitive, and emotional change.

The results obtained when evaluating the intervention programs, on the one hand, ratify many of the hypotheses proposed and, on the other hand, are consistent with various investigations carried out on children’s play (Lillard 2017; Russ and Schafer 2006). In the same direction as other research, these studies confirm the positive role of play, especially friendly, cooperative, and creative play, in child development and intragroup relationships within the school context. Recent research that has used adapted versions of the interventions presented in the present study has shown us how children can develop creative thinking and more positive emotional states (Celume et al. 2019b) and can deploy various emotional competencies, such as the identification of emotions and collaboration (Celume et al. 2020). The successive works carried out validate the programs designed and provide a tool for psychological interventions based on cooperative-creative play to develop personality during childhood. They emphasize the importance of including structured ludic experiences in the school curriculum to promote communication, empathy, prosocial behavior (behaviors of helping, cooperating, sharing), and creativity and prevent violence.

## Figures and Tables

**Table 1:** Effects of cooperative game programs on child development.

Results. Cooperative Play Stimulates:	Studies
Spontaneous cooperative behavior in different contexts	Orlick et al. (1978); Orlick and Foley (1979)
Participation in sharing behaviors	Orlick (1981)
Cooperative social responses. Motor variables (spatial orientation, eye-hand coordination, ability to throw, run, jump, hit) and, especially, static and dynamic balance	Mender et al. (1982)
Cooperative interactions in class, self-acceptance and acceptance of others, participation in class activities, and a positive classroom environment	Blazic (1986)
Positive physical and verbal contact during free play, decreasing negative physical contact and negative verbal interactions	Grineski (1989)
The social interaction and motor development of children of all groups, with greater influence on children with visual problems	Zanandrea (1998)
Helping behaviors, cooperation, the ability to incorporate others, and group cohesion	Carlson (1999)
Reasons for others’ feelings and the capacity for empathy	Brownell et al. (2002); Celume et al. (2020)
A decrease in peer rejection in the classroom context	Mikami et al. (2005)
Prosocial behaviors, decreasing the dissocial ones in schoolchildren with problems of coexistence	Beltrán (2007)
The level of collaboration, the time they spent cooperating, and the depth with which they collaborated	van der Aalsvoort and van der Leeden (2009)
Prosociality in schoolchildren institutionalized due to high psychosocial risk, reducing direct and indirect aggressiveness	Valencia (2010)
Adjusted cooperative motor behaviors	Lavega et al. (2014)
Psychomotor domain (motor performance, postural tonic control, schema and body image, coordination of arms and legs) and social skills	Cuesta Cañadas et al. (2016)
An increase of acceptance in dyads and a decrease in the number of isolated students	Andueza and Lavega (2017)
More intense positive emotions	Miralles et al. (2017)
Self-esteem	Vega (2018)
Prosocial behaviors in physical education classes	Navarro-Patón et al. (2019)
A decrease in aggressive behaviors	Haro (2019)
Social skills	Guevara and Ubillus (2019); Ochoa (2019); Guerreros (2021); Goldstein and Lerner (2018)
Social interaction, rule-making and enforcement, constructive conflict management, and participation in promoting the common good	Garcia (2021)

**Table 2:** Variables and evaluation instruments of the early childhood education program (preschool) (Garaigordobil 2007).

Variables Evaluated	Assessment Instruments
Cognitive strategies for prosocial interpersonal problem-solving	Interpersonal problem-solving test (Garaigordobil and Berrueco 2007b)
Altruism: sharing with peers and adults	Assessment of altruism (Leighton 1992a, 1992b)
Intelligence: verbal, non-verbal, and total	Brief Intelligence Test (Kaufman and Kaufman [1994] 1997)
Neuropsychological maturity factors: comprehensive language, visoperception, iconic memory, verbal fluency, and attention	Child Neuropsychological Maturity Questionnaire (Portellano et al. 2000)
Development factors: affective reaction, somatic development, sensory development, motor reaction, sensorimotor coordination, contact and communication, conceptualization, and normativity	Observational Scale of Development (parents) (Secadas [1988] 1992)
Creative personality behaviors and traits	Scale of Creative Personality Behaviors and Traits (parents and teachers) (Garaigordobil and Berrueco 2007c)
Verbal creativity: fluency, flexibility, and originality. Graphic-figurative creativity: fluency, originality, abreaction, and elaboration	Creative Thinking Test: Verbal and graphic battery Torrance ([1974] 1990)
Creative thinking: attention to detail, nonconformity, and identifying and solving problems	Creative thinking test by image analysis (Garaigordobil and Berrueco 2007d)

**Table 3:** Program variables and assessment instruments for children aged 6 to 8 years (Garaigordobil 2005a).

Variables Evaluated	Assessment Instruments
Social behaviors: leadership, joviality, social sensitivity, respect-self-control, aggressiveness-stubbornness, apathy-withdrawal, anxiety-shyness, and social adaptation	Socialization Battery (teachers) (Silva and Martorell 1983)
Image of the group companions: playmate (sociograms)	Sociometric Questionnaire (peers) (Garaigordobil 2005e)
Capacity for group cooperation	Evaluation of group cooperation: The Game of Squares (Garaigordobil 2005f)
Self-concept	Child Self-Concept Assessment Scale (Garaigordobil 2005g)
Emotional stability	The Human Figure Drawing Test (Koppitz 1976)
Body schema	Body Schema Recognition Test (Garaigordobil 2005h)
Maturity aptitudes for school learning: verbal comprehension, numerical aptitude, perceptual aptitude, global maturity for learning	Battery of Aptitudes for School Learning (De-la-Cruz 1982)

**Table 4:** Program variables and assessment instruments for children aged 8 to 10 years (Garaigordobil 2003b).

Variables Evaluated	Assessment Instruments
Non-altruistic behavior	Experimental technique for evaluating altruistic behavior: The Prisoner’s Dilemma (adaptation Garaigordobil 2003c)
Social behavior: assertive, passive, and aggressive	Assertive Behavior Scale for Children (Wood et al. 1983)
Social behavior: helping behaviors	Socialization Battery. Social Sensitivity Scale (teachers) (Silva and Martorell 1983)
Intra-group communication: positive and negative messages	Evaluation of intra-group communication: The Game of Silhouettes (peers) (Garaigordobil 2003d)
Self-concept: bodily, intellectual, social, and emotional	List of Adjectives to evaluate self-concept (Garaigordobil 2003e)
Verbal creativity: fluency, flexibility, and originality	Verbal Tasks of the Guilford Battery (Guilford 1951)
Graphic creativity: fluidity, flexibility, originality, and connectivity. Graphic creativity: abreaction, originality, fantasy, and connectivity	Graphic tasks of the Guilford Battery (Guilford 1951) Abreaction Test to Evaluate Creativity (De-la-Torre 1991)

**Table 5:** Program variables and assessment instruments for children aged 10 to 12 years (Garaigordobil 2004a).

Variables Evaluated	Assessment Instruments
Social behavior: assertive, passive, and aggressive	Assertive Behavior Scale for Children (Wood et al. 1983)
Antisocial and criminal behavior	Antisocial-Delinquent Behavior Questionnaire (Seisdedos [1988] 1995)
Social behaviors: consideration for others, leadership, self-control, withdrawal, and anxiety	Socialization Battery (self-assessment) (Silva and Martorell 1989)
Prosocial behavior	Prosocial Behavior Questionnaire (teachers and parents) (Weir and Duveen 1981)
Prosocial and creative classmates	Sociometric Questionnaire: prosocial and creative classmate (peers) (Garaigordobil 2004b)
Cognitive strategies for the resolution of social situations: Assertive, aggressive, and passive	Cognitive Strategies for the Resolution of Social Situations Questionnaire (Garaigordobil 2004c)
Self-concept: positive and negative (total) and creative	Lists of adjectives to evaluate the global and creative self-concept (Garaigordobil 2004d)
Emotional stability	The Human Figure Drawing Test (Koppitz 1976)
Intelligence: verbal, non-verbal, and Total	Brief Intelligence Test (Kaufman and Kaufman [1994] 1997)
Verbal associative thinking (verbal creativity): fluidity and originality	Word association test (Garaigordobil 2004f)
Creative personality behaviors and traits	Creative Personality Scale (self-assessment, parents, and teachers) (Garaigordobil 2004e)
Verbal creativity: fluency, flexibility, and originality. Graphic creativity: abstraction title, abreaction, fluency, flexibility, and originality	Torrance Creative Thinking Test (Torrance [1974] 1990)
Graphic creativity: performance time and originality of a graphic task	Free creation of a picture. Inter-judge evaluation of a creative product (Garaigordobil 2004g)

**Table 6:** Means, standard deviations, and results of the analysis of variance of the experimental and control groups in all the measured variables in the pretest and posttest phase and the pretest-posttest difference. Evaluation of the Early Childhood Education Game Program (preschool, 4–6 years).

	Experimental Group (*n* = 53)						Control Group (*n* = 33)						Experimental—Control (*n* = 86)			
	Pretest		Posttest		Pre-Pos		Pretest		Posttest		Pre-Pos		ANOVAF (1, 84)		ANCOVAF (1, 84)	Cohen’s *d*
	*M*	*SD*	*M*	*SD*	*M*	*SD*	*M*	*SD*	*M*	*SD*	*M*	*SD*	Pretest	Posttest	Posttest	
Prosocial interpersonal problem-solving	8.25	3.58	11.21	3.86	2.96	4.86	8.03	4.05	9.03	3.71	1.00	5.12	0.06	6.66 *	6.52 *	0.39
Altruistic behavior																
With adults	1.24	1.27	1.63	1.39	0.39	1.46	1.58	1.15	1.94	1.18	0.35	1.33	1.52	1.06	0.22	0.02
With peers	2.84	2.20	3.92	1.84	1.08	2.90	2.55	1.52	2.77	1.36	0.23	1.87	0.42	9.02 **	8.87 **	0.34
Intelligence																
Verbal intelligence	23.55	5.86	27.98	4.99	4.43	3.46	24.21	3.97	27.39	3.55	3.18	2.44	0.33	0.34	3.01 +	0.41
Nonverbal intelligence	15.40	3.43	18.00	3.70	2.60	3.75	15.18	3.19	17.36	3.76	2.18	4.22	0.08	0.59	1.04	0.10
Global intelligence	38.94	8.36	45.98	6.95	7.04	5.01	39.39	5.37	44.76	6.34	5.36	4.57	0.07	0.67	3.80 +	0.35
Neuropsychological																
Attention	8.79	3.90	10.96	4.10	2.17	4.68	7.76	2.73	10.88	4.04	3.12	4.06	1.77	0.00	0.04	0.21
Verbal fluency	14.09	7.56	20.81	9.24	6.72	11.84	13.85	7.31	14.24	7.15	0.39	9.01	0.02	12.12 ***	14.75 ***	0.60
Comprehensive language	4.75	2.34	5.62	1.91	0.87	2.16	5.48	2.11	6.09	1.77	0.61	1.94	2.13	1.28	0.34	0.12
Iconic memory	5.94	1.88	6.96	1.43	1.02	2.00	5.97	1.31	6.76	1.50	0.79	1.52	0.00	0.40	0.20	0.12
Visual perception	8.21	3.04	11.94	2.41	3.74	2.43	8.79	3.19	11.76	2.67	2.97	2.04	0.71	0.11	1.11	0.34
Scale of development																
Conceptualization	12.46	2.30	13.92	1.89	1.46	1.84	12.18	2.31	14.11	2.18	1.93	1.84	0.27	0.15	0.01	0.25
Contact-communication	10.25	1.87	10.94	1.55	0.69	1.58	10.39	2.04	11.18	1.85	0.79	1.55	0.10	0.36	0.13	0.06
Sensorimotor coordination	6.48	1.21	7.27	0.93	0.79	1.09	6.86	1.18	7.18	1.02	0.32	0.90	1.78	0.16	2.28	0.47
Somatic development	5.60	1.67	5.90	1.74	0.31	1.42	5.54	1.86	5.54	1.53	0.00	1.81	0.02	0.88	1.02	0.19
Sensory arousal	1.94	0.24	2.00	0.00	0.06	0.24	1.89	0.31	1.93	0.26	0.03	0.33	0.63	3.90	3.49	0.10
Normativity	3.40	0.72	3.85	0.36	0.44	0.75	3.18	0.94	3.46	0.64	0.29	1.08	1.42	11.67 ***	10.73 ***	0.16
Affective reaction	4.77	1.32	5.17	1.45	0.40	1.33	4.86	1.53	4.61	1.57	−0.25	1.92	0.07	2.61	3.78 +	0.39
Motor reaction	3.77	1.55	4.85	1.59	1.08	1.43	3.75	1.67	4.54	1.32	0.79	1.45	0.00	0.77	1.16	0.20
Creative personality																
Parent evaluation	28.25	5.84	29.53	6.18	1.27	4.99	27.80	6.16	28.20	6.32	0.40	5.80	0.06	0.53	0.07	0.16
Teacher evaluation	23.96	7.01	26.94	6.32	2.98	4.02	18.40	4.78	19.73	7.11	1.33	3.77	8.26 **	14.24 ***	5.06 *	0.42
Verbal creativity																
Flexibility	15.92	4.23	23.66	5.78	7.74	6.47	14.70	4.86	15.39	3.89	0.70	4.16	1.52	52.54 ***	46.00 ***	1.29
Fluency	25.47	8.42	41.77	13.88	16.30	13.22	22.00	9.03	24.55	9.13	2.55	8.61	3.27 +	39.99 ***	32.82 ***	1.23
Originality	29.34	15.45	58.23	30.33	28.89	30.34	25.79	18.81	26.52	17.55	0.73	14.80	0.90	29.77 ***	23.09 ***	1.17
Graphic creativity																
Abreaction	6.83	4.00	7.98	3.89	1.15	4.48	8.52	5.04	7.42	4.14	−1.09	4.85	2.94 +	0.39	3.06 +	0.47
Elaboration	16.13	8.09	29.60	9.61	13.47	7.36	19.00	9.89	21.09	8.03	2.09	8.88	2.15	18.02 ***	38.27 ***	1.39
Fluency	5.64	4.16	12.55	5.10	6.91	5.00	6.97	4.93	8.36	4.33	1.39	5.47	1.79	15.31 ***	24.97 ***	1.05
Originality	7.94	7.52	19.06	9.80	11.11	9.16	8.79	8.10	10.94	8.52	2.15	9.91	0.24	15.38 ***	25.29 ***	0.93
Creative thinking																
Attention to detail	17.51	5.14	24.21	3.81	6.70	4.56	19.12	4.45	21.30	4.02	2.18	5.62	2.20	11.31 ***	14.85 ***	0.88
Nonconformity	1.51	1.37	1.45	1.26	−0.06	1.46	1.73	1.07	0.76	1.00	−0.97	1.19	0.60	7.16 **	7.92 **	0.68
Fluency	8.21	3.22	13.79	4.84	5.58	4.70	7.79	3.40	8.18	3.45	0.39	4.33	0.33	33.61 ***	35.83 ***	1.14
Originality	4.49	3.95	11.13	8.85	6.64	8.07	4.18	5.29	4.45	4.37	0.27	6.62	0.09	16.26 ***	18.31***	0.86

+ *p* < 0.09 * *p* < 0.05 ** *p* < 0.01 *** *p* < 0.001.

**Table 7:** Means, standard deviations, and results of the analysis of variance of the experimental and control groups in all the measured variables in the pretest and posttest phase and the pretest-posttest difference. Evaluation of the Game program 6–8 years.

	Experimental Group (*n* = 125)						Control Group (*n* = 53)						Experimental—Control (*n* = 178)			
	Pretest		Posttest		Pre-Pos		Pretest		Posttest		Pre-Pos		ANOVAF (1, 176)		ANCOVAF (1, 176)	Cohen’s *d*
	*M*	*SD*	*M*	*SD*	*M*	*SD*	*M*	*SD*	*M*	*SD*	*M*	*SD*	Pretest	Posttest	Posttest	
Social behavior																
Leadership	19.84	14.07	30.70	11.98	10.86	9.78	20.57	11.87	23.45	12.33	2.89	11.34	0.10	13.36 ***	33.01 ***	0.75
Joviality	25.63	7.07	29.61	4.81	3.98	5.68	26.09	7.53	26.15	6.12	0.06	6.47	0.15	16.24 ***	18.38 ***	0.64
Social sensitivity	13.82	8.52	25.14	9.58	11.32	10.13	15.87	9.20	18.64	7.82	2.77	9.23	2.05	18.97 ***	31.83 ***	0.88
Respect for self-control	37.54	10.85	43.18	10.27	5.65	9.68	38.53	13.60	38.08	14.80	−0.45	11.86	0.26	6.98 **	12.36 ***	0.56
Aggressiveness	7.73	8.21	4.88	6.29	−2.85	6.92	5.23	7.48	8.53	10.12	3.30	8.20	3.63 +	8.51 **	18.50 ***	0.81
Apathy withdrawal	9.14	10.90	3.14	5.02	−6.00	8.78	4.42	7.69	4.96	6.73	0.55	5.33	8.20 **	3.98 *	22.75 ***	0.90
Anxiety shyness	11.38	8.75	6.99	6.61	−4.38	6.92	4.38	5.25	6.42	4.40	2.04	4.68	29.36 ***	0.33	7.33 **	1.08
Social adjustment	33.43	6.64	38.01	4.68	4.58	5.06	34.02	7.26	34.32	7.39	0.30	5.03	0.27	16.02 ***	31.76 ***	0.84
Group cooperation																
Task performance time	9.96	6.06	1.60	0.70	−8.36	6.02	8.82	4.77	6.27	3.58	−2.55	4.39	0.30	40.45 ***	35.98 ***	1.10
Giving-receiving behaviors	1.09	0.90	5.30	2.61	4.20	2.79	1.22	0.72	1.66	1.13	0.44	1.20	0.16	19.45 ***	32.33 ***	1.75
Asking-receiving behaviors	0.25	0.25	1.80	2.45	1.55	2.46	0.43	0.30	0.25	0.22	−0.17	0.39	3.61 +	4.26 *	3.31 +	0.97
Helping behaviors	0.81	0.69	2.44	1.75	1.62	1.92	0.41	0.46	1.21	1.07	0.80	1.13	3.13 +	4.58 *	4.55 *	0.52
Refusing to give	0.47	0.40	0.46	0.72	−0.01	0.93	0.64	0.59	0.40	0.60	−0.24	0.94	1.68	0.01	0.10	0.24
Taking away	1.70	1.64	1.02	1.67	0.68	2.09	1.02	0.91	1.10	1.56	−0.08	1.54	0.99	0.06	0.59	0.49
Self-concept	20.88	4.15	22.91	2.85	2.03	3.93	20.70	4.26	21.62	3.47	0.92	4.28	0.07	6.67 **	6.97 **	0.27
Emotional instability	1.88	1.60	0.97	1.03	−0.91	1.49	2.38	1.50	1.87	1.26	−0.51	1.58	3.73 +	24.79 ***	20.5 ***	0.26
Body schema	14.40	3.57	19.00	2.12	4.60	3.19	15.92	4.45	18.77	2.92	2.85	3.48	5.83 *	0.33	4.69 *	0.52
School learning																
Verbal comprehension	13.40	3.25	16.34	2.09	2.94	2.81	15.17	2.95	16.21	2.67	1.04	2.19	11.67 ***	0.11	7.48 **	0.75
Numerical aptitude	11.75	4.03	15.60	2.98	3.85	2.95	13.42	4.25	15.68	3.34	2.26	2.86	6.14 *	0.02	4.87 *	0.54
Perceptual aptitude	37.26	8.58	43.24	5.67	5.98	6.63	37.58	7.65	42.32	6.45	4.74	8.65	0.05	0.90	2.97 +	0.16
Maturity index	62.41	13.20	75.18	8.93	12.77	9.24	66.17	10.48	74.21	9.75	8.04	9.78	3.39 +	0.41	7.26 **	0.49

+ *p* < 0.09 * *p* < 0.05 ** *p* < 0.01 *** *p* < 0.001.

**Table 8:** Means, standard deviations, and results of the analysis of variance of the experimental and control groups in all the measured variables. in the pretest and posttest phase and the pretest-posttest difference. Evaluation of the GAME program 8–10 years.

	Experimental Group (*n* = 126)						Control Group (*n* = 28)						Experimental—Control (*n* = 154)			
	Pretest		Posttest		Pre-Pos		Pretest		Posttest		Pre-Pos		ANOVAF (1, 152)		ANCOVAF (1, 152)	Cohen’s *d*
	*M*	*SD*	*M*	*SD*	*M*	*SD*	*M*	*SD*	*M*	*SD*	*M*	*SD*	Pretest	Posttest	Posttest	
Non-altruistic behavior	23.28	6.83	13.43	5.72	−9.84	7.81	23.60	4.96	22.40	7.45	−1.20	5.64	0.02	26.41 ***	20.53 ***	1.26
Social behavior																
Aggressive behavior	11.21	8.98	4.12	3.81	−7.08	7.82	6.74	4.95	4.07	3.52	−2.66	4.01	6.24 *	0.01	3.56 +	0.71
Passive behavior	7.71	4.73	6.91	3.46	−0.79	4.54	6.81	3.90	9.25	4.56	2.44	4.83	0.83	11.11 ***	19.68 ***	0.68
Non-assertive behavior	18.92	8.89	11.01	5.18	−7.91	6.76	13.55	6.94	13.33	5.75	−0.22	5.72	8.67 **	5.05 *	26.64 ***	1.22
Social behavior																
Helping behaviors	15.47	4.54	18.16	4.54	2.69	3.53	20.22	4.51	20.48	5.19	0.25	4.38	24.26 ***	6.44 *	1.08	0.61
Communication																
Positive messages	14.04	8.18	27.75	8.90	13.90	10.23	9.34	2.92	8.57	2.88	−0.86	3.97	8.60 **	126.31 ***	109.41 ***	1.90
Negative messages	4.56	4.30	0.24	0.57	−4.31	4.24	2.07	1.96	2.71	2.55	0.64	2.32	8.87 **	98.58 ***	87.50 ***	1.07
Self-concept																
Intellectual self-concept	1.71	0.92	2.22	0.74	0.51	0.95	1.46	0.96	1.93	1.01	0.46	1.31	1.64	3.07 +	2.02	0.04
Body self-concept	3.94	1.47	4.33	1.16	0.40	1.16	4.04	1.29	3.96	1.34	−0.07	1.63	0.10	2.16	3.64 +	0.33
Affective self-concept	6.56	2.71	7.96	2.16	1.40	2.15	6.75	2.13	7.04	2.23	0.29	2.74	0.12	4.13 *	6.50 *	0.45
Social self-concept	4.67	2.34	6.90	1.12	2.24	2.11	4.71	2.22	5.21	2.00	0.50	2.68	0.01	37.19 ***	42.06 ***	0.72
Global self-concept	16.87	6.24	21.42	3.86	4.55	4.62	16.96	4.24	18.14	5.21	1.18	6.09	0.00	14.35 ***	21.05 ***	0.62
Verbal creativity																
Fluency uses	4.02	2.27	10.83	6.02	6.80	5.71	4.29	1.88	6.50	3.01	2.21	2.76	0.32	13.62 ***	13.20 ***	1.02
Flexibility uses	2.55	1.25	5.94	2.54	3.40	2.73	3.21	1.19	4.00	1.76	0.79	1.66	6.61*	14.74 ***	14.76 ***	1.15
Originality uses	1.90	2.50	9.24	6.99	7.33	6.99	2.68	2.46	4.71	4.14	2.04	4.68	2.20	10.82 ***	10.03 **	0.88
Verbal creativity																
Fluency consequences	3.19	2.05	7.54	3.17	4.35	3.60	3.50	1.40	3.89	1.31	0.39	1.68	0.57	35.61 ***	36.43 ***	1.40
Flexibility consequences	2.25	1.17	4.20	1.70	1.95	1.76	2.10	1.03	2.60	1.13	0.50	1.23	0.53	22.41 ***	22.33 ***	0.95
Originality consequences	1.00	2.16	5.27	4.66	4.27	4.67	1.67	2.95	1.78	2.26	0.11	3.03	0.48	14.85 ***	15.58 ***	1.05
Graphic creativity																
Fluency circles	14.66	5.24	29.20	9.64	14.54	9.10	13.03	6.60	21.50	7.78	8.46	7.68	2.00	15.59 ***	20.34 ***	0.72
Flexibility circles	7.04	1.87	9.47	2.18	2.42	2.68	6.50	2.70	8.28	1.74	1.78	2.88	1.64	7.28 **	6.48 *	0.23
Originality circles	4.09	3.83	11.33	7.85	7.23	7.75	5.25	4.87	8.10	5.65	2.85	5.89	1.87	4.22 *	6.61 *	0.63
Connectivity circles	3.66	3.29	13.65	9.04	9.98	8.85	4.17	5.39	7.92	6.86	3.75	6.70	0.42	9.91 **	14.38 ***	0.79
Graphic creativity																
Abreaction	15.09	6.46	17.61	5.02	2.52	7.47	14.92	7.30	13.46	3.73	−1.46	7.06	0.01	17.00 ***	19.63 ***	0.54
Originality	9.90	5.63	13.72	6.03	3.81	7.35	8.17	5.41	8.64	4.61	0.46	6.77	2.18	17.52 ***	12.78 ***	0.47
Fantasy	1.45	2.47	2.55	2.57	1.10	3.08	2.21	2.31	0.78	1.37	−1.42	2.28	2.21	12.39 ***	14.09 ***	0.92
Connectivity	1.16	4.53	2.92	5.64	1.76	7.22	0.00	0.00	0.53	2.00	0.53	2.00	1.84	4.86 *	5.14 *	0.23

+ *p* < 0.09 * *p* < 0.05 ** *p* < 0.01 *** *p* < 0.001.

**Table 9:** Means, standard deviations, and results of the analysis of variance of the experimental and control groups in all the measured variables in the pretest and posttest phase and the pretest-posttest difference. Evaluation of the Game program 10–12 years.

	Experimental Group (*n* = 54)						Control Group (*n* = 32)						Experimental—Control (*n* = 86)			
	Pretest		Posttest		Pre-Pos		Pretest		Posttest		Pre-Pos		ANOVAF (1, 84)		ANCOVAF (1, 84)	Cohen’s *d*
	*M*	*SD*	*M*	*SD*	*M*	*SD*	*M*	*SD*	*M*	*SD*	*M*	*SD*	Pretest	Posttest	Posttest	
Social behavior																
Passive behavior	8.39	6.11	7.17	6.18	−1.22	5.76	7.97	5.40	6.22	4.22	−1.75	5.33	0.10	0.58	1.26	0.09
Assertive behavior	16.91	5.04	18.98	4.70	2.07	4.30	18.00	5.46	18.03	5.43	0.03	4.83	0.88	0.73	1.94	0.44
Aggressive behavior	4.54	4.60	2.74	3.11	−1.80	3.24	4.00	4.17	5.13	6.01	1.12	3.87	0.29	5.87 *	14.43 ***	0.81
Social behavior																
Antisocial behavior	3.39	3.40	3.24	2.63	−0.15	3.76	3.62	2.54	5.31	4.22	1.69	2.91	0.11	7.88 **	6.70 *	0.54
Delinquent behavior	0.41	1.46	0.15	0.36	−0.26	1.39	0.06	0.25	1.06	2.69	1.00	2.70	1.74	6.11 *	5.33 *	0.58
Social behavior																
Consideration behaviors	29.44	7.08	32.44	5.71	3.00	6.19	27.66	6.20	30.19	6.24	2.53	5.48	1.40	2.92 +	1.51	0.08
Self-control behaviors	30.52	5.36	31.96	6.32	1.44	6.81	29.03	5.44	27.12	5.90	−1.91	4.21	1.52	12.37 ***	8.41 **	0.59
Withdrawal behaviors	6.85	5.08	5.48	5.37	−1.37	4.97	6.16	6.34	5.06	5.30	−1.09	5.43	0.31	0.12	0.16	0.05
Anxiety behaviors	9.28	4.58	7.57	5.47	−1.70	5.01	9.16	4.10	9.03	5.34	−0.13	3.91	0.01	1.45	1.90	0.34
Leadership behaviors	17.04	5.27	20.26	5.75	3.22	4.80	15.00	4.98	16.38	6.12	1.38	4.78	3.12 +	8.73 **	6.14 *	0.38
Prosocial behavior																
Teacher evaluation	39.11	14.08	42.43	12.57	3.31	13.20	39.37	11.81	41.26	9.58	2.42	7.26	0.00	0.20	0.17	0.08
Parent evaluation	41.22	8.86	46.46	7.28	5.24	7.99	40.50	8.82	41.00	10.81	0.61	7.34	0.13	7.72 **	10.49 **	0.60
Social behavior																
Prosocial peer	3.69	3.07	6.07	3.32	2.39	2.26	3.50	2.78	4.81	2.61	1.31	1.82	0.07	3.38 +	5.96 *	0.52
Interaction strategies																
Assertive	2.39	1.64	4.69	2.21	2.30	2.02	2.47	2.06	3.09	1.96	0.63	2.03	0.03	11.29 ***	10.57 **	0.82
Aggressive	5.02	2.33	2.35	1.97	−2.67	2.74	3.63	1.77	1.97	1.87	−1.66	1.98	8.52 **	0.78	0.00	0.42
Passive	1.50	1.50	3.39	1.98	1.89	1.95	0.91	1.20	1.56	1.37	0.66	1.72	3.62 +	21.26 ***	10.09 **	0.66
Total	8.91	2.90	10.43	4.01	1.52	3.64	7.00	1.92	6.62	3.06	−0.38	3.36	10.95 ***	21.33 ***	11.31 ***	0.54
Self-concept																
Positive	29.39	6.23	31.66	6.36	2.42	6.80	26.75	7.53	27.06	7.19	0.31	6.87	3.08 +	9.44 **	7.61 **	0.30
Negative	3.78	4.59	3.06	2.52	−0.42	4.08	2.56	2.51	3.97	2.98	1.41	2.42	1.90	2.27	2.44	0.54
Total	25.61	8.28	28.60	6.68	2.83	7.48	24.19	8.25	23.09	7.69	−1.09	7.35	0.59	12.10 ***	10.94 ***	0.52
Creative	13.81	3.78	15.17	3.87	1.47	3.73	12.72	3.54	12.69	4.25	−0.03	3.85	1.77	7.61 **	5.54 *	0.39
Emotional instability	3.58	2.01	1.45	1.41	−2.13	2.32	2.78	1.54	2.34	1.91	−0.44	2.00	3.76	6.07 *	7.94 **	0.78
Intelligence																
Vocabulary	36.98	3.48	40.22	4.96	3.24	5.53	36.48	3.41	38.58	3.26	2.10	2.64	0.40	2.71	0.61	0.26
Definitions	10.37	3.42	13.43	4.49	3.06	3.43	8.06	3.03	10.13	3.66	2.06	3.35	9.69 **	12.08 ***	4.49 *	0.29
Verbal intelligence IQ	47.35	5.48	53.65	6.60	6.30	5.56	44.55	5.66	48.71	5.81	4.16	3.35	5.03 *	11.99 ***	4.83 *	0.46
Nonverbal intelligence IQ	30.98	4.89	33.35	5.50	2.37	5.53	30.58	5.31	33.65	6.22	3.06	4.77	0.12	0.05	2.80	0.13
Total intelligence IQ	78.33	8.94	87.00	10.56	8.67	8.55	75.13	9.24	82.35	10.57	7.23	6.50	2.46	3.81 *	0.10	0.18
Associative thinking																
Fluency	23.93	12.29	30.69	13.03	6.76	14.50	19.91	9.63	24.44	6.72	4.53	9.75	2.50	6.33 *	4.06 *	0.18
Originality	21.76	19.27	35.17	23.25	13.41	24.36	13.25	11.38	20.87	9.81	7.62	13.64	5.15 *	10.90 ***	6.87 **	0.29
Creative personality																
Self-assessment	39.00	10.72	47.35	9.63	8.35	9.68	35.69	7.29	39.47	9.92	3.20	8.54	2.39	12.66 ***	10.39 **	0.56
Parent evaluation	36.69	10.32	39.41	9.53	2.72	6.98	35.78	8.85	37.07	10.20	1.33	5.35	0.17	1.10	1.53	0.22
Teacher evaluation	28.65	12.81	38.13	10.47	9.48	11.93	34.00	10.53	35.67	8.90	1.90	6.04	3.98 *	1.84	5.20 *	0.80
Creative behavior																
Creative peer	3.57	2.60	4.93	3.63	1.35	3.12	3.34	2.86	2.91	2.67	−0.44	2.59	0.14	7.48 **	8.91 **	0.62
Verbal creativity																
Fluency	20.72	9.21	34.57	16.38	13.85	16.35	23.66	8.86	34.38	14.08	10.72	13.37	2.09	0.00	0.30	0.20
Originality	11.72	9.91	39.93	21.02	28.20	21.74	14.59	9.11	27.69	15.15	13.09	13.47	1.78	8.28 **	12.14 ***	0.83
Flexibility	13.39	4.68	18.83	5.73	5.44	5.67	13.25	4.20	17.88	4.79	4.62	5.25	0.01	0.63	1.56	0.15
Graphic creativity																
Abstraction title	0.91	0.73	1.28	0.88	0.37	1.15	1.50	0.80	1.41	0.67	−0.09	1.00	12.21 ***	0.51	0.00	0.42
Abreaction	12.96	3.58	16.33	2.45	3.37	3.94	13.72	3.27	12.94	2.71	−0.78	3.90	0.95	35.65 ***	39.71 ***	1.05
Originality	8.63	6.89	12.89	6.69	4.26	7.41	7.50	3.57	9.53	5.57	2.03	5.53	0.74	5.70 *	5.45 *	0.34
Elaboration	5.06	2.62	13.67	5.13	8.61	4.62	7.50	3.50	10.87	4.54	3.38	4.95	13.54 ***	6.46*	13.22 ***	1.09
Fluency	22.87	7.40	25.52	6.47	2.65	6.63	20.22	4.90	25.44	5.78	5.22	5.05	3.25 +	0.00	0.16	0.43
Graphic creativity: picture																
Time	14.56	3.34	12.54	3.41	−2.02	3.94	14.41	5.40	17.94	5.97	3.53	5.58	0.02	28.63 ***	36.23 ***	1.14
Originality																
Evaluator 1	2.20	1.11	3.09	1.14	0.89	1.46	2.38	1.31	2.22	1.31	0.16	1.55	0.41	10.56 **	11.88 ***	0.69
Evaluator 2	2.54	1.28	2.98	1.12	0.44	1.28	2.53	1.32	2.38	1.21	−0.16	1.71	0.00	5.51 *	5.97 *	0.39

+ *p* < 0.09 * *p* < 0.05 ** *p* < 0.01 *** *p* < 0.001.

## Data Availability

Data are not publicly available.
